# Ophthalmology

**Published:** 1878-12

**Authors:** 


### Ophthalmology.

Retinal Photo-Chemistry. (*Br. Med. Journ.*, Aug. 31, 1878.)

In 1870 Boll discovered that the retina is only absolutely colorless and transparent for two-thirds the depth of its lowest layer, and that for the lowest third of the rods it is plunged in a purple-colored substance; this coloration having been long unknown, because the mere access of light destroys it, decolorizing it with extreme rapidity. The study of this substance—the retinal purple—and its modifications by light, led the author to these formal conclusions: That the action exercised upon the retina by light is of a chemical order, and the formation of images a true photography. Very positive images of objects before which the eyes have been exposed immediately before and after death, put this fact beyond contest. Light effaces and destroys this retinal purple, but it is physiologically re-secreted during life in proportion as it is decolorized.

Soon after these experiments and their verification, Kühne sought and discovered the organ of the incessant reproduction of this retinal purple. This organ is the mosaic layer, or hexagonal epithelium of the choroid, which must now be definitely attached to the retina itself, under the name already proposed by several anatomists, of retinal epithelium.

M. Giraud-Teulon, in a recent report to the Académie de Médecine, in which he discusses the new state of facts, develops the new considerations which it introduces into the pathological theory of the production of color. Thus, so far as concerns the persistence of positive images—that is to say, the survival of the sensation after the impression which has produced it—the very fact of the chemical decoloration of the retinal purple by light, implying a certain time for its reconstitution—or the secretory regeneration of it by the hexagonal layer—sufficiently accounts for the greater or less persistance of the image.

As to the accidental negative images and their successive colored phases, the perfectly arbitrary explanation of the three orders of fibers, of Young, is naturally supplanted by the following mechanism. A given monochromatic light chemically alters in a constant and uniform manner the retinal purple which it encounters. The rod or primitive nervous element plunges its foot into the bath formed by this substance. The whole hypothesis is, therefore, limited to admitting that this nerve element possesses the faculty of being affected in a different manner by the intimate contact of different media, just as is the case with the papillae of the nerves of special sensation ; just as the gustatory and olfactory nerves, for instance, appreciate or carry to the sensorium incitations as varied as the nature of the liquid or effluvia which attack their expansions. Inversely, when the primary cause—the luminous object—has been removed, the nerve fiber, in proportion as the chemical reconstitution of the retinal purple proceeds, testifies to the gradual revivification of the normal bath.

M. Giraud-Teulon concludes by indicating the resources which pathological physiology will find in the photo-chemical theory of vision, for the reformation of the theory of Daltonism, and the explanation of other normal and morbid phenomena in the history of colored entoptic sensations.

This "vision red," or purple, or *erythopsin*, as its discover names it, attains its maximum after a night's rest or sleep, or when an animal has been kept for some hours in darkness ; it is soluble in solutions of the biliary acids and in glycerine ; and probably plays a part in the red reflection from the fundus of the eye seen in ophthalmoscopic examinations of the eye.

---

